# SARS-CoV-2 and risk of psychiatric hospital admission and use of psychopharmaceuticals: A nationwide registry study of 4,585,083 adult Danish citizens

**DOI:** 10.1192/j.eurpsy.2023.2418

**Published:** 2023-06-07

**Authors:** Valdemar Rømer, Pradeesh Sivapalan, Josefin Eklöf, Susanne D. Nielsen, Zitta B. Harboe, Tor Biering-Sørensen, Theis Itenov, Jens-Ulrik S. Jensen

**Affiliations:** 1 Section of Respiratory Medicine, Herlev-Gentofte University Hospital, Hellerup, Denmark; 2 Department of Infectious Diseases, University Hospital of Copenhagen, Copenhagen, Denmark; 3 Department of Pulmonary and Infectious Diseases, University Hospital of Copenhagen, North Zealand, Denmark; 4 Department of Cardiology, Herlev-Gentofte University Hospital, Hellerup, Denmark; 5 Centre for Health and Infectious Diseases Research (CHIP), University Hospital of Copenhagen, Copenhagen, Denmark; 6 Department of Anaesthesiology and Intensive Care, University Hospital of Copenhagen, Copenhagen, Denmark; 7 Department of Clinical Medicine, Faculty of Health Sciences, University of Copenhagen, Copenhagen, Denmark

**Keywords:** Long COVID, psychiatry, psychopharmaceuticals, SARS-CoV-2

## Abstract

**Background:**

Current evidence on the risk of admission- or medication-requiring psychiatric sequelae of severe acute respiratory syndrome coronavirus 2 (SARS-CoV-2) infection is limited to selected populations, short durations, and loss to follow-up. This study examined if SARS-CoV-2 infection was associated with increased long-term risk of psychiatric admissions and *de novo* prescription of psychoactive medication in the general population of Denmark.

**Methods:**

Adults (≥18 years) were assigned to either the control or SARS-CoV-2 group based on polymerase chain reaction (PCR) tests between 1 January 2020 and 27 November 2021. Infected subjects were matched 1:5 to control subjects by propensity score. Incidence rate ratios (IRRs) were calculated. Adjusted Cox regression was applied to the unmatched population with SARS-CoV-2 infection as a time-dependent covariate. Follow-up time was 12 months or until the end of the study.

**Results:**

A total of 4,585,083 adults were included in the study. Approximately 342,084 had a PCR-confirmed SARS-CoV-2 infection and were matched 1:5 with 1,697,680 controls. The IRR for psychiatric admission was 0.79 in the matched population (95% confidence interval [CI]: 0.73–0.85, *p* < 0.001). In the unmatched population, the adjusted hazard ratios (aHR) for psychiatric admission were either below 1.00 or with a 95% CI lower limit of 1.01. SARS-CoV-2 infection was associated with an increased risk of *de novo* prescription of psychoactive medication in both the matched (IRR 1.06, 95% CI: 1.02–1.11, *p* < 0.01) and unmatched population (HR 1.31, 95% CI: 1.28–1.34, *p* < 0.001).

**Conclusions:**

We found a signal of increased use of psychoactive medication, specifically benzodiazepines, among SARS-CoV-2-positive persons, but the risk of psychiatric admissions did not increase.

## Introduction

It is estimated that more than 1 in 10 with acute COVID-19 experience symptoms persisting after the primary severe acute respiratory syndrome coronavirus 2 (SARS-CoV-2) infection – commonly known as long COVID or post-COVID conditions [1, 2]. The symptoms reported vary from chest pain, fatigue, dyspnea, coughing, cognitive impairment, psychological distress, and several others [3–5].

A retrospective cohort study from the United States found an association between coronavirus disease-2019 (COVID-19) and an increased risk of first psychiatric diagnosis (anxiety was the most prevalent) within 14 to 90 days post-infection compared to the risk of psychiatric diagnoses after other infections such as influenza [6]. Other studies also reported an increased occurrence of neurological and psychiatric disorders or altered mental state, where most psychiatric diagnoses were new/first diagnoses [7–9]. For the aforementioned studies, the follow-up time was relatively short, ranging from 2 to 24 weeks, which could have been a weakness, since psychiatric sequelae may take some time to develop [10]. There are little data on mental health in adults recovering from COVID-19, especially in those with symptoms weeks to months after their initial infection or Long COVID [6].

It has been speculated whether SARS-CoV-2 infection could influence mental outcomes via a biological link: the first case of suspected SARS-CoV-2 meningitis was seen in February 2020 [11]. Since then, it has been established that SARS-CoV-2 infection is associated with several neurobiological outcomes, resulting from either hyperinflammatory or hypercoagulable states, direct central nervous system (CNS) infection, and postinfectious immune-mediated processes [12–15]. Other coronaviruses have been shown to have such potential [16, 17]. Furthermore, it has been suggested that these impacts on the CNS could be linked to psychiatric sequelae of SARS-CoV-2 infection [14], although conflicting evidence on inflammation and symptom severity of some psychiatric disorders has been reported [18].

Outcomes of SARS-CoV-2 infection on mental health are poorly investigated [19–21]. Understanding potential implications of SARS-CoV-2 infection on mental health to preempt psychiatric events is important. Based on the diverging results from small studies, with somewhat selected populations and with considerable loss to follow-up, more solid data are needed from studies taking these limitations into account.

In this study, which is a nationwide cohort study with all adults in our country, with accurate information on the timing of polymerase chain reaction (PCR) positivity of SARS-CoV-2 virus, and complete follow-up, we sought to investigate whether a first positive SARS-CoV-2 PCR test is associated with an increased risk of psychiatric admission or prescription of psychoactive medication as compared to uninfected controls.

## Methods

We conducted a nationwide retrospective population-based registry study utilizing the National Danish registries. The study was approved by the Danish Data Protection Agency (j.no. P-2021-360). In Denmark, informed consent is not required for retrospective studies.

### Hypotheses

1. SARS-CoV-2 infection increases the risk of being admitted to a psychiatric hospital department.
2. SARS-CoV-2 infection increases the risk of *de novo* prescription of psychoactive medication.

### Data sources

All persons in Denmark are assigned a unique personal identification number, which is used in all national registers, enabling accurate linkage between them [22]. The following registries were used:

1. The Danish Central Personal Registry containing person identification number, sex, vital status, and death date of all persons residing in Denmark since 1968 [22]
2. The Danish National Patient Registry containing data on all somatic hospital contacts nationwide since 1977, including all psychiatric hospital contacts since 1994 [23]
3. The National Prescription Registry containing data on dispensed prescriptions since 2004 [24]
4. The Danish Microbiology Database containing data on PCR-confirmed SARS-CoV-2 infection since February 2020 [25].
5. The Danish Vaccination Registry containing data on vaccinations since 1996 [26].

We did not have data from general practitioners; thus, diagnoses were derived from hospital registries. Reliable data on the indications for prescribed medications were not available. We did not have data on the specialization of the prescriber. Data on income and postal code were not obtained. During the study period, frequent PCR test for COVID-19 was encouraged and freely available regardless of symptoms. The PCR laboratory data did only contain one instance per person being either the first SARS-CoV-2-positive test, if any, or the last SARS-CoV-2 negative test, and did not contain information on symptoms or severity of disease.

### Study population

The population was defined as all adults (18 years or older) residing in Denmark (except Greenland and Faroe Islands) by January 1, 2020. The only exclusion criteria were invalid SARS-CoV-2 PCR test date data (e.g., test registered postmortem) to prevent persons at risk from not being eligible – thus including persons with an ongoing psychiatric hospitalization at the beginning of the study. SARS-CoV-2 infection was defined by a registered positive PCR test nonregarding symptoms, severity of disease, and hospitalization. The first registered SARS-CoV-2 infection in Denmark was on February 26, 2020 [27]. The study period was defined as January 1, 2020 to November 27, 2021 to report pre-pandemic baseline values and omit interference with the omicron-variant of which the first registered case in Denmark was November 28, 2021 [27]. Comorbidities were defined by a hospital contact or outpatient clinic contact with an active diagnosis within 5 years before baseline and classified using the Charlson Comorbidity Index (CCI) [28]. Medication at baseline was defined by ≥1 collected prescription within 1 year prior to baseline.

A *propensity score-matched model* was used in the primary analysis, comparing the SARS-CoV-2 infected to matched controls to assess the risk of reaching an endpoint between similar groups. Follow-up for SARS-CoV-2-infected subjects started by the date of infection. Follow-up for controls was started by the same date as their matched infected subject, or they were excluded if dead at the time of starting follow-up. Follow-up for all individuals in the primary analysis was 12 months or until the end of the study.

To further test the findings of the primary analysis, and to also provide a true nationwide perspective, a secondary analysis of the complete, unmatched population was followed by January 1, 2020. All persons started as controls; SARS-CoV-2 infection was treated as a time-dependent variable allowing persons to change status during the study from noninfected to infected. Subjects were followed until the end of the study or 12 months postinfection.

### Outcomes

The primary outcome was psychiatric hospital admissions, defined as psychiatric hospital contacts of more than 24 hours initiated at least one calendar-date after inclusion with International Classification of Diseases, Tenth Revision (ICD-10) codes F-20 to F-50 as the primary diagnosis. Transfers from somatic to psychiatric units were also considered for this outcome. This selection of diagnoses includes affective disorders, anxiety disorders, and psychotic disorders, but not, e.g., personality disorders, attention deficit hyperactivity disorder, and mental retardation, as we could not reasonably suspect a causal association between SARS-CoV-2 infection and these.

The secondary outcome was the *de novo* prescription of any psychoactive medication, regardless of indication, as we did not have data on this. However, assuming the somatic indications are infrequent compared to the psychiatric indications when not hospitalized, the risk ratio estimates should still be reliable as an approximate measure for medication-requiring psychiatric conditions. *De novo* psychoactive medication was considered a categorical variable (either none or at least one prescription). It was assumed that most persons in active psychopharmacological treatment would collect their prescribed medication in intervals of less than 3 months. As we did not have data on whether a prescription collection was due to the commencement or continuation of treatment, individuals with a prescription collection within 90 days were excluded from the analysis to increase the likelihood that events were true *de novo* prescriptions. Psychoactive medication was defined by the Anatomical Therapeutic Chemical (ATC) codes for antidepressants (N06A), benzodiazepines and benzodiazepine-like drugs (N03AE, N05BA, N05CD, N05CF), antipsychotics (N05A except N05AN01), and lithium (N05AN01).

In the case of a positive SARS-CoV-2 PCR test on the calendar date of admission in the time-dependent Cox-regression on the unmatched population, this was considered an event happening while noninfected, as to avoid the outcome psychiatric hospital admission itself increasing the risk of an event and a short time-to-event in the SARS-COV-2-infected group.

The proportion of persons vaccinated against COVID-19 in the different groups was reported, including the proportion in the SARS-CoV-2 group vaccinated ≥14 days before infection.

### Statistical analysis

Continuous variables were presented as mean values with 95% confidence intervals (CIs) or medians with interquartile range (IQR). Categorical variables were presented as proportions.

In the *propensity score-matched population*, the control sample was created utilizing a parallel balanced propensity score-matched model using the nearest neighbor greedy-match algorithm from the MatchIt package (R v. 4.1.3) [29]. SARS-CoV-2 infected subjects were matched 1:5 to controls. Matching was performed on age, sex, CCI score, baseline history of psychoactive medication, and baseline history of hospital contacts with registered psychiatric diagnoses in order to achieve presumed equal-risk groups at baseline. Outcomes were reported as incidence rates (IR) and incidence rate ratios (IRRs) with 95% CI.

We analyzed the *unmatched population* using a Cox proportional hazard regression model with SARS-CoV-2 infection as a time-dependent covariate. Calendar time was accounted for in the regression model as all participants were followed from the same calendar date (1 January 2020). The Cox model was tested for the proportional hazards assumption and linearity. In addition, the model was adjusted for age, sex, and CCI score. In this multivariate regression on the total unmatched population, it was decided not to adjust for baseline psychiatric history to avoid overfitting and removal of relevant differences between groups. Outcomes were reported as hazard ratios (HR) and adjusted hazard ratios (aHR) with 95% CI and *p*-values.

Interaction analyses on psychiatric admission was performed using the two-way analysis of variance (ANOVA) test on Cox regressions comparing additive to multiplicative models [30]. Interaction was tested between [SARS-CoV-2 infection status] (time-dependent) and independent variables with suspected interaction and highest main effect:

- [SARS-CoV-2 Alpha versus Delta variant community-domination] (time-dependent)
- [age ≥ 40 years versus age < 40 years] (baseline)
- [male versus female]
- [COVID-19 vaccination ≥14 days before infection yes versus no]

Variant community-domination time periods were chosen for interaction-testing as different social restrictions and risk of severe infection if infected in this time periods differed and could plausibly explain more than infection with SARS-CoV-2 during any period itself, as disease severity was different between variants [27, 31]. SARS-CoV-2 Alpha variant community domination was defined as the period from the beginning of study until 4 July 2021. The delta variant dominated from 5 July 2021 until the end of the study (the study was terminated before any registered SARS-CoV-2 Omicron variant infections in Denmark) based on evidence from the Danish health authorities (Statens Serum Institut) [27]. Age was suspected to increase the risk of psychiatric sequelae following infection. A review of COVID-19 sequalae reports mean ages of patients with symptoms post-infection from 43 to 63 years [32] and a study on self-reported sequelae of SARS-CoV-2 in different age groups found that all groups with age ≥ 40 years was associated with increased odds of sequelae compared to all groups with age < 40 years [33], why this was chosen as the cut-off value in the interaction-test. Further, it can be suspected that the effect of the SARS-CoV-2 infection and the associated induced social isolation and disease on the risk of psychiatric admission differs between this group and people of older age in a non-additive manner. Sex was included in the interaction analysis as characteristics between males and females admitted to a psychiatric department varies [34], and as was sex suspected to influence the susceptibility to psychiatric sequelae of COVID-19 [34, 35]. It was decided to test interaction for vaccination against COVID-19 as this suspected interaction seems relevant and plausible however unsure; infections ≥14 days post-vaccination have been associated with a reduced risk of psychotic disorder (but not mood and anxiety disorders) [36] and reduced odds of long COVID (however with an upper limit of the 95% CI of 0.99) [37]. When an interaction was found, outcomes were presented separately for interacting variables.

## Results

**Figure 1.**
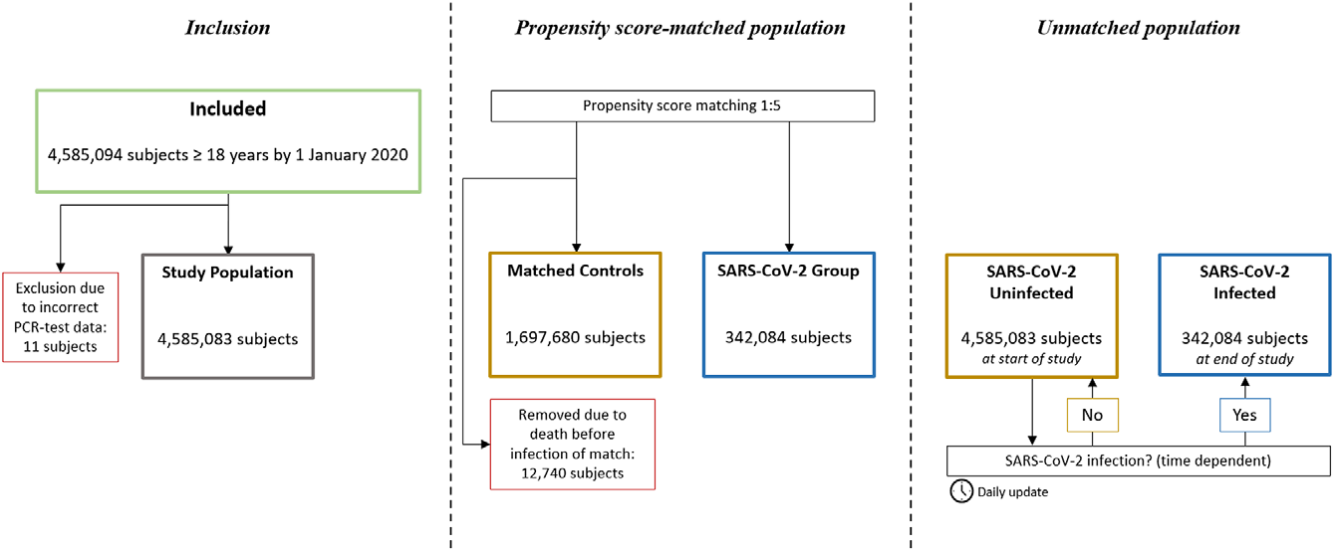
Study flowchart. In the *propensity score-matched population*, subjects in the SARS-CoV-2 Group and their corresponding matches were observed from the date of the registered positive PCR-test, or removed if dead by then. In the *unmatched population*, all subjects were observed from 1 January 2020, and SARS-CoV-2 infection was treated as a time dependent variable.

A total of 4,585,094 individuals aged ≥18 years were identified. In total, 11 individuals were excluded due to incorrect registration date of SARS-CoV-2 PCR test, leaving a total study population of 4,585,083 subjects. Of these, 342,084 were registered with a confirmed positive SARS-CoV-2 PCR test. In the *propensity score-matched population*, all the SARS-CoV-2 positive individuals were matched 1:5 with 1,697,680 controls (12,740 controls were removed due to death before infection of match) (Figure 1). No subjects were lost to follow-up.

The baseline values of those infected with SARS-CoV-2 differed from the total population. The infected were generally younger, less medicated with psychoactive medication, and had fewer comorbidities. Baseline values were overall similar in the SARS-CoV-2 infected and controls (Table 1).

**Table 1.** Baseline patient demographic and clinical characteristics by 1 January 2020

Characteristics	Unmatched	Propensity score-matched population	
	Total adult population (*N* = 4,585,083)	SARS-CoV-2 infected (*N* = 342,084)	Matched controls (*N* = 1,697,680)
Age, median (IQR)	49 (33 to 65)	40 (27 to 54)	40 (27 to 53)
Male sex, *n* (%)	2,256,635 (49.22)	167,674 (49.02)	832,363 (49.03)
History of psychiatric admission			
≥1 psychiatric admission within 1 year, *n* (%)	18,561 (0.40)	878 (0.26)	5,451 (0.32)
*Depression*	3,701 (0.08)	202 (0.06)	986 (0.06)
*Anxiety disorder*	1,419 (0.03)	99 (0.03)	443 (0.03)
*Bipolar or mania*	2,011 (0.04)	98 (0.03)	530 (0.03)
*Schizophrenia or psychosis*	7,491 (0.16)	267 (0.08)	2,197 (0.13)
Medication			
Prescribed psychoactive medication, *n* (%)	594,093 (12.96)	31,827 (9.30)	155,203 (9.14)
*Antidepressants*	400,905 (8.74)	22,067 (6.45)	108,315 (6.38)
*BZD and BZD-like*	230,009 (5.02)	11,365 (3.32)	52,811 (3.11)
*Antipsychotics*	115,765 (2.52)	5,673 (1.66)	32,175 (1.90)
*Lithium*	8,962 (0.20)	417 (0.12)	2,479 (0.15)
Comorbidities			
Specialist treated psychiatric illness, *n* (%)	233,253 (5.09)	16,123 (4.71)	77,806 (4.58)
*Depression*	81,448 (1.78)	5,529 (1.62)	24,835 (1.46)
*Anxiety disorder*	64,487 (1.41)	4,709 (1.38)	22,953 (1.35)
*Bipolar*	15,550 (0.34)	776 (0.23)	4,682 (0.28)
*Schizophrenia*	27,603 (0.60)	1,162 (0.34)	8,750 (0.52)
COPD, *n* (%)	148,711 (3.24)	9,635 (2.82)	39,529 (2.33)
Diabetes Mellitus, *n* (%)	140,826 (3.07)	8,297 (2.43)	34,145 (2.01)
Stroke and transient cerebral ischemia, *n* (%)	90,266 (1.97)	3,925 (1.15)	18,744 (1.10)
Charlson Comorbidity Index, mean (CI)	1.18 (1.18 to 1.18)	0.68 (0.67 to 0.68)	0.64 (0.64 to 0.64)

Abbreviations: CI, 95% confidence interval; COPD, chronic obstructive lung disease; IQR, interquartile range.

The proportion receiving at least one vaccine against COVID-19 varied between groups. In the total population, 4,096,800 (89.25%) received at least one vaccine during the study. Among the SARS-CoV-2 infected, this number was 273,095 (79.83%), and 1,515,930 (89.29%) for the matched controls. In the SARS-CoV-2 group, 82,921 (24.24%) were vaccinated ≥14 days before infection.

### Propensity score-matched population

The incidence rate of psychiatric admission was 360 per 100,000 subjects in the SARS-CoV-2 infected group, and 460 per 100,000 person-years among the matched controls. This corresponds to an IRR of 0.79 (95% CI 0.73 to 0.85, *p* < 0.001), thus SARS-CoV-2 infection was not associated with an increased risk of psychiatric admission.

Stratifying for vaccination against COVID-19 ≥ 14 days before infection or start of observation of controls, the IRR of psychiatric admission did not relevantly differ between the vaccinated (IRR 0.77, 95% CI 0.55 to 1.08, *p* = 0.13) and the unvaccinated (IRR 0.79, 95% CI 0.72 to 0.86, *p* < 0.001).

**Figure 2.**
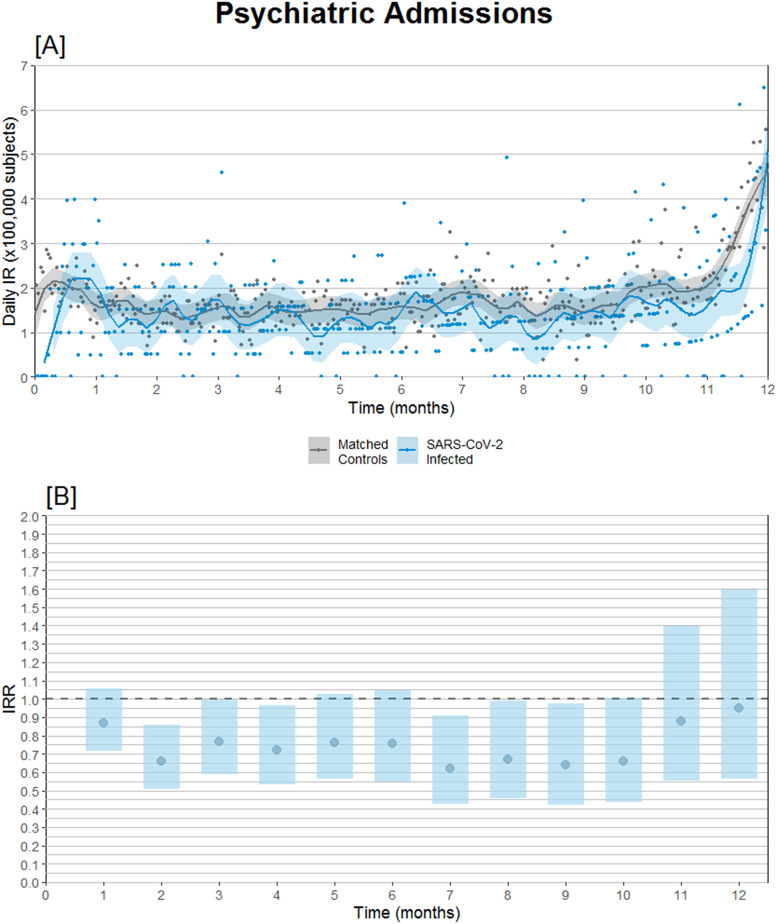
Psychiatric hospital admission incidence comparison after SARS-CoV-2 infection compared to propensity score-matched controls observed from the same date as the infected match. (A) Daily psychiatric admission incidence rates. (B) Psychiatric admission incidence rate ratios (IRRs) with 95% confidence intervals.

Subdividing admissions by primary diagnosis of admission, reduced incidence of admission with schizophrenia or psychosis seems to be the main driver of the overall reduced IRR, whereas admissions coded with either depression, anxiety disorders, and bipolar/mania were neutral (Table 2). Furthermore, the relative risk of psychiatric admission was lowest during the second-month postinfection (Figure 2B).

**Table 2.** Incidence rates and incidence rate ratios of psychiatric outcomes after SARS-CoV-2 infection compared to propensity score-matched controls observed from the same date as the infected match

	Matched controls (*N* = 1,697,680)	SARS-CoV-2 infected (*N* = 342,084)	*p*-value
Follow-up			
Lost to follow-up, *n* (%)	0/2,687,120 (0%)	0/342,084 (0%)	
Psychiatric admission			
Admitted within 12 months, IR × 10^5^	459.63	360.42	
Admitted within 12 months, IRR (CI)	Ref.	0.79 (0.73 to 0.85)	<0.001
*Depression*	Ref.	0.92 (0.78 to 1.09)	0.36
*Anxiety disorders*	Ref.	1.03 (0.81 to 1.31)	0.82
*Bipolar or manias*	Ref.	1.07 (0.86 to 1.32)	0.55
*Schizophrenia or psychosis*	Ref.	0.64 (0.56 to 0.74)	<0.001
Psychoactive medication			
*De novo* prescription within 12 months, IR × 10^5^	1,424	1,512	
*De novo* prescription within 12 months, IRR (CI)	Ref.	1.06 (1.02 to 1.11)	<0.01
*Antidepressants*	Ref.	1.04 (0.99 to 1.09)	0.17
*BZD and BZD-like*	Ref.	1.17 (1.10 to 1.25)	<0.001
*Antipsychotics*	Ref.	0.92 (0.82 to 1.03)	0.14
*Lithium*	Ref.	0.97 (0.53 to 1.75)	0.91

*Note: De novo* prescription of psychoactive medication was defined as the first new prescription of any psychoactive medication, excluding subjects with a history of psychoactive medication within 3 months prior to start of observation.

Abbreviations: BZD, benzodiazepines; CI, 95% confidence interval; IR, incidence rate; IRR, incidence rate ratio.

SARS-CoV-2 infection was associated with an increased risk of *de novo* prescription of psychoactive medication was found (IRR 1.06, CI 1.02 to 1.11, *p* < 0.001). When granulating the results for type of psychoactive medication, use of benzodiazepines and benzodiazepine-like medication seemed to be a driver of the signal: IRR of 1.17 (CI 1.10 to 1.25, *p* < 0.001) (Table 2).

### Unmatched population

#### Interaction analysis

Interaction analysis was performed on *psychiatric hospital admission* for variables [SARS-CoV-2 infection status] and, respectively, (i) [age < 40 years versus age ≥ 40 years] (*p* < 0.001), (ii) sex [male versus female] (*p* = 0.12), (iii) [SARS-CoV-2 Alpha versus Delta variant community-domination] (*p* = 0.01) and (iv) [COVID-19 vaccination ≥14 days before infection yes versus no] (*p* = 0.06). As a result of this, HR were presented separately for all combinations of interacting variables (i) and (iii).

#### Hazard estimates

SARS-CoV-2 infection was not associated with an increased risk of psychiatric hospital admission in the age group <40 years, neither in the Alpha variant community-dominance period (aHR 0.79, CI 0.75 to 0.82, *p* < 0.001) nor in the Delta variant community-dominance period (aHR 0.67, CI 0.56 to 0.80, *p* < 0.001).

In the age group ≥40 years, SARS-CoV-2 infection was not associated with an increased risk of psychiatric hospital admission in the Alpha variant community-dominance period (aHR 0.96, CI 0.87 to 1.06, *p* = 0.38). However, for this age group, the Delta variant community-dominance period was associated with an increased risk of psychiatric hospital admission (aHR 1.34, 1.01 to 1.77, *p* = 0.04).

**Figure 3.**
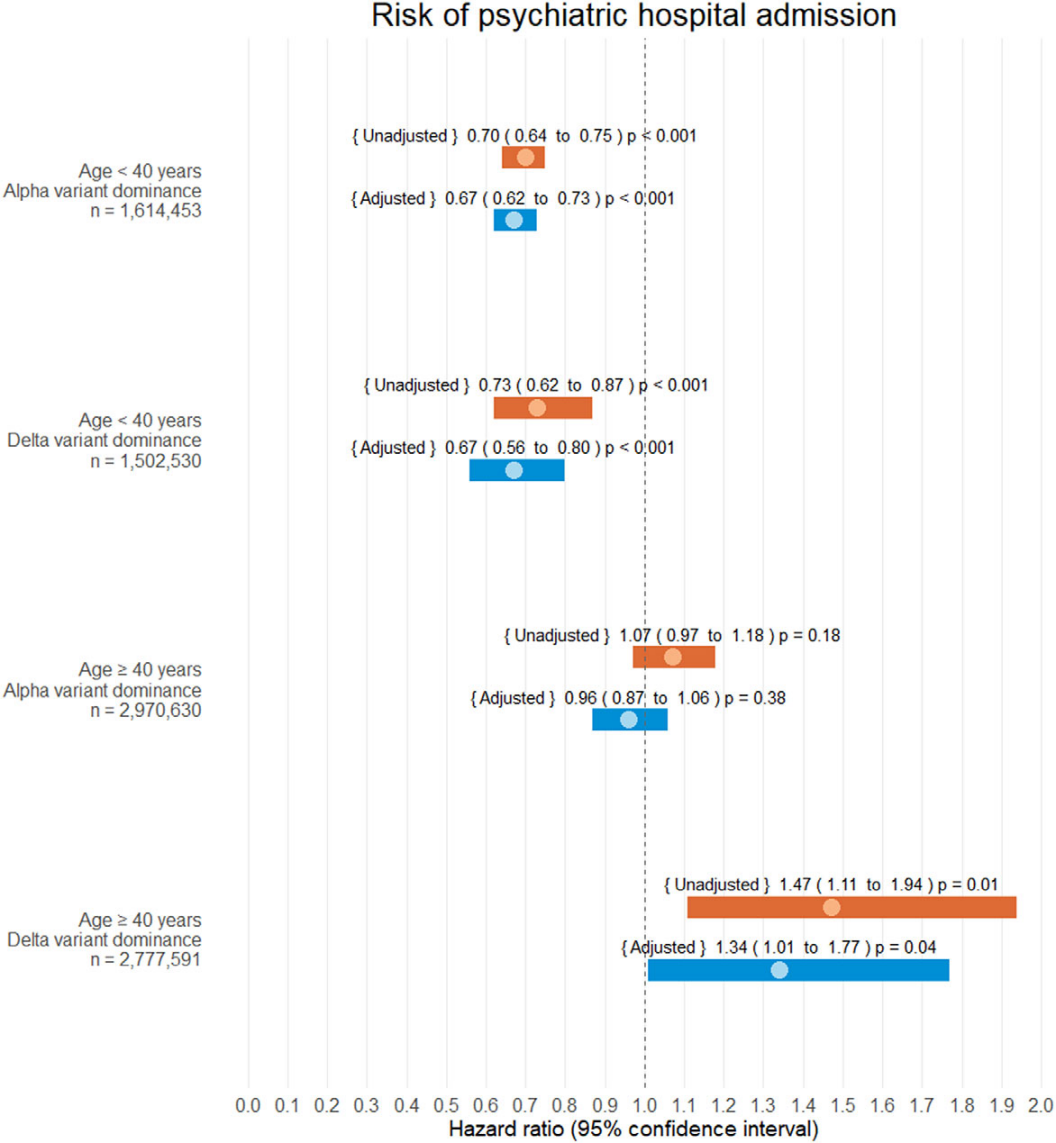
Hazard ratio estimates of psychiatric hospital admission following SARS-CoV-2 infection. Infection and alpha/delta variant dominance were treated as time-dependent covariates. During the study 342,084 of 4,585,083 subjects were infected with SARS-CoV-2 (7.46%). No patients were lost to follow-up.

Refer to Figure 3 for all adjusted and unadjusted HR estimates on psychiatric admission.

SARS-CoV-2 was associated with an increased risk of a *de novo* prescription of any psychoactive pharmaceutical (aHR 1.31, CI 1.28 to 1.34, *p* < 0.001). An association with increased risk of *de novo* prescription of all subgroups of psychoactive medication, except lithium, was found (Table 3:).

**Table 3.** Hazard ratio estimates for *de novo* prescription of psychoactive medication after SARS-CoV-2 infection in the unmatched population

	Total population (*N* = 4,585,083)	*p*-value
*Follow-up*		
Lost to follow-up, *n*/*N* (%)	0/4,585,083 (0.00)	
SARS-CoV-2 infected during study, *n*/*N* (%)	342,084/4,585,083 (7.46)	
*Psychoactive medication*		
*De novo* prescription within 12 months, unadjusted HR (CI)	1.12 (1.10 to 1.15)	<0.001
Adjusted^a^ HR (CI)	1.31 (1.28 to 1.34)	<0.001
*Antidepressants*	1.09 (1.06 to 1.12)	<0.01
*BZD and BZD-like*	1.65 (1.60 to 1.71)	<0.001
*Antipsychotics*	1.05 (1.00 to 1.11)	<0.05
*Lithium*	1.20 (0.88 to 1.63)	0.24

*Note*: Infection was treated as a time-dependent covariate.

Abbreviations: BZD, benzodiazepines; CI, 95% confidence interval; HR, hazard ratio.

a Adjusted for age, sex, and Charlson’s Comorbidity Index score.

Except for the apparent increased risk of *de novo* prescription of antidepressant and antipsychotic drugs for the unmatched population, all other estimates were similar to the risk estimates obtained from the propensity score-matched population.

## Discussion

In this nationwide registry-based cohort study investigating whether PCR-confirmed SARS-CoV-2 infection is associated with psychiatric hospital admission and prescription of psychoactive medication, we found no clinically relevant associations between SARS-CoV-2 infection and increased risk of psychiatric admission, neither in the propensity score-matched population nor in the unmatched population. Vaccination against COVID-19 did not seem to alter this risk.

Generally, SARS-CoV-2 infection was not or only slightly associated with an increased risk of *de novo* prescription of any psychoactive medication. We found an increased risk of *de novo* prescription of benzodiazepines and benzodiazepine-like pharmaceuticals in the propensity score matched model and the adjusted Cox regression, but no other clinically relevant elevated risks were found analyzing sub-groups of psychoactive medication. Benzodiazepines are used for a wide range of conditions, including acute anxiety, alcohol- and substance-withdrawal, insomnia, delirium, and prior to medical, dental, or surgical procedures. According to the Danish national guidelines, the first choice for pharmacological treatment of anxiety disorders is antidepressants (selective serotonin reuptake inhibitors specifically) [38]; however, benzodiazepines still seem to be considered a good quick-onset short-term treatment option by many clinicians [39] why this association with SARS-CoV-2 and increased risk of benzodiazepines/benzodiazepine like pharmaceuticals could be interpreted as an increased long-term risk of acute pathological non-hospital-requiring anxiety possibly in combination with insomnia and alcohol- or substance-withdrawal. As the prescriber and indication for prescribed psychoactive medication was not known, it is not possible to conclude neither if the increased benzodiazepine treatment was specialist-requiring nor the proportions of the risk estimate driven by psychiatric and somatic indication.

Risk estimates should not be overinterpreted, as they are likely influenced by unmeasured confounding, possibly that individuals infected with SARS-CoV-2 could have a less “anxious” mindset resulting in exposure to the infection. Schizophrenia was the most common primary diagnosis of psychiatric admission; however, these patients were underrepresented in the SARS-CoV-2 group, and propensity score matching did not fully account for this difference. This could explain the unexpected protective risk estimate of SARS-CoV-2 on hospital admission with schizophrenia or psychosis as the primary diagnosis. Also, as there was a general reduction in psychiatric hospitalizations in Denmark during the pandemic [40], it is likely that psychiatric hospital admission-requiring individuals were either not admitted at all or hospitalized for severe COVID-19 in a somatic hospital to ensure isolation until recovered from SARS-CoV-2 infection, thus not registered as a psychiatric admission by diagnosis code, unless transferred after discontinuation of isolation and COVID-19 treatment.

Only a few studies on long-term risk of prescription of psychoactive medication after SARS-CoV-2 infection exist; a retrospective registry-based study of American veterans with a similar study design (prescription based, propensity score matching and synchronized inclusion dates of infected and controls) showed an increased risk of antidepressant use (HR 1.55) and use of benzodiazepines (HR 1.65) [41]. However, the higher HR reported could be explained at least in part by the homogeneity (all military veterans, 89% males, 81% overweight or obese, 59% current or former smokers) and high mean age (63 years) of the American veteran cohort. Therefore, it seems very plausible that the selected cohort was more vulnerable to psychiatric events. This study better represents a general adult population as we included all Danish adults with no exceptions or further selection.

The outcomes of this study not signaling increased risk of psychiatric sequelae challenge the signals and interpretation of other studies. A large systematic review on hospitalized COVID-19 patients reported both short and long-term (up to 7 months after discharge) psychiatric sequelae such as depression and anxiety [42]. However, most included studies did not have a control group or compared only hospitalized COVID-19 patients to non-hospitalized healthy controls. These studies do not provide knowledge on mental health sequelae of SARS-CoV-2 infection but only of severe COVID-19 requiring hospitalization. The ANCHOHVID study, an Andalusian prospective cohort study, did not detect any difference in mental health-related sequelae after discharge between patients hospitalized for COVID-19 and patients hospitalized for other causes [43]. This supports the findings of the present study that SARS-CoV-2 infection itself does not seem to be associated with an increased long-term risk of severe psychiatric sequelae. It can be speculated that psychiatric sequelae are instead a more general characteristic of severe disease and hospital admission.

A major strength of this study is that we followed the total Danish adult population above 18 years of age (4.5 million individuals). This is important, as previous studies based on hospital-data and smaller selected databases are not representative of the general population; opposite, this study very precisely represents reality of the broad general population not excluding non-hospital requiring SARS-CoV-2 infection. We had a longer follow-up than most studies investigating psychiatric sequalae of SARS-CoV-2 infection (12 months). No subjects were lost to follow-up and we had access to validated and complete data on hospital admissions and prescriptions. PCR tests were free of charge and widely available. Regular testing for SARS-CoV-2 was encouraged in periods with high transmission rates, and a negative test was required in many settings such as restaurant visits and museums. Access to treatment and hospitalization is free of charge for all Danish residents, thus bias due to avoidance of proper psychiatric treatment for economic reasons can be ruled out. Targeted treatment of COVID-19 with monoclonal antibodies was not standard of care during the study period, so effective treatment of COVID-19 cannot be assumed to have influenced the outcomes.

There are some limitations to this study. There were major differences in baseline between the uninfected and the infected in the total population. Although we did perform both adjusting and matching, in sequence, there may have been some residual confounding, possibly including income, social class, and geography. The lower vaccination rate in the SARS-CoV-2 infected group compared to the matched controls could indicate differences in socio-demographic factors such as a lower household income and lower level of education among the unvaccinated [44]. Further, in a similar population, a lower COVID-19 vaccination rate has been reported among the psychiatric vulnerable – despite being offered vaccination earlier than the general population [45], thus possibly affecting risk estimates in an unknown direction. Despite PCR confirmation being encouraged after a positive SARS-CoV-2 antigen test, there is a risk some might not have PCR-confirmed the diagnosis, and thus be included in the non-infected group although actually being infected. Different test patterns in vulnerable groups could affect risk estimates: according to a study on an overlapping population and time period, psychiatric vulnerable persons had lower odds of PCR testing than the general population [46]. We did not have access to SARS-CoV-2 variant data; hence the distribution of variants among the infected is unknown, which could have affected the outcomes and comparability of the findings to other studies. It is however known that only the alpha and delta variants have been community dominant during the study period [27], and as we stratified for this on the primary outcome in the Cox regression, we do not consider this a major limitation. As the delta variant only dominated for approximately 4 months, this could explain the wide confidence interval of the hazard estimate for this strata, and possibly part of the apparent increase in hazard among the infected during this period if the ratio of hazards was not truly constant over time. We did not have information on the indication for the prescriptions of psychoactive medication; psychoactive medication is prescribed for a variety of conditions like insomnia, delirium, and pain. Also, it cannot be ruled out that some events of *de novo* prescription of psychoactive medication were not true commencements of treatment, as the period chosen was arbitrary and could not account for all discontinuations before the beginning of the study – neither that some events were actually continued treatment in persons with collection pick-up frequency of more than 90 days.

In conclusion, this is the first study to investigate the impact of SARS-CoV-2 infection on psychiatric outcomes in an entire country, with access to precise data on test positivity and complete follow-up. Psychiatric admissions did not increase among the SARS-CoV-2 infected. However, the risk of prescription of psychoactive medication, specifically benzodiazepines, seemed to increase.

Possibly, our results reflect a moderately increased risk of mild-to-moderate anxiety-spectrum disorders among the SARS-CoV-2 positive.

We believe our results should lead to close monitoring of psychiatric sequalae in future pandemics of SARS-CoV-2 and other infectious viruses in the general population, as it seems post-acute treatment-requiring anxiety-related psychiatric manifestations origin may occur in a broader population regardless of symptom severity.

## Data Availability

We believe that knowledge sharing increases the quantity and quality of scientific results. Sharing of relevant data will be discussed within the study group upon reasonable request.
